# Simulation Analysis of Equibiaxial Tension Tests for Rubber-like Materials

**DOI:** 10.3390/polym15173561

**Published:** 2023-08-27

**Authors:** Huaan Luo, Yinlong Zhu, Haifeng Zhao, Luqiang Ma, Jingjing Zhang

**Affiliations:** 1 School of Intelligent Manufacturing, Nanjing Vocational College of Information Technology, Nanjing 210023, China; 2 Jiangsu Robot Micro Servo Engineering Research Center, Nanjing 210023, China; 3 College of Mechanical and Electronic Engineering, Nanjing Forestry University, Nanjing 210037, China

**Keywords:** hyperelasticity, equibiaxial tension, EAP, inflation tension, radial tension, equibiaxial planar tension

## Abstract

For rubber-like materials, there are three popular methods of equibiaxial tension available: inflation tension, equibiaxial planar tension, and radial tension. However, no studies have addressed the accuracy and comparability of these tests. In this work, we model the tension tests for a hyperelastic electroactive polymer (EAP) membrane material using finite element method (FEM) and investigate their experimental accuracy. This study also analyzes the impact of apparatus structure parameters and specimen dimensions on experimental performances. Additionally, a tensile efficiency is proposed to assess non-uniform deformation in equibiaxial planar tension and radial tension tests. The sample points for calculating deformation in inflation tensions should be taken near the top of the inflated balloon to obtain a more accurate characteristic curve; the deformation simulation range will be constrained by the material model and its parameters within a specific limit (*λ* ≈ 1.9); if the inflation hole size is halved, the required air pressure must be doubled to maintain equivalent stress and strain values, resulting in a reduction in half in inflation height and decreased accuracy. The equibiaxial planar tension test can enhance uniform deformation and reduce stress errors to as low as 2.1% (at *λ* = 4) with single-corner-point tension. For circular diaphragm specimens in radial tension tests, increasing the number of cuts and using larger punched holes results in more uniform deformation and less stress error, with a minimum value of 3.83% achieved for a specimen with 24 cuts and a 5 mm punched hole. In terms of tensile efficiency, increasing the number of tensile points in the equibiaxial planar tension test can improve it; under radial tension, increasing the number of cuts and decreasing the diameter of the punched hole on the specimen has a hedging effect. The findings of this study are valuable for accurately evaluating various equibiaxial tension methods and analyzing their precision, as well as providing sound guidance for the effective design of testing apparatus and test plans.

## 1. Introduction

**Figure 1:**
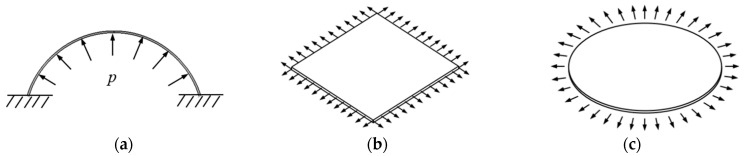
Typical equibiaxial tension test methods: (**a**) inflation tension; (**b**) equibiaxial planar tension; and (**c**) radial tension.

Because friction between contact surfaces during uniaxial compression tests for rubber-like materials causes complicated stress states such as compression and shear, resulting in inaccurate results, equibiaxial tension tests are commonly used to replace compression tests for rubber-like materials and have recently gained popularity for assessing the mechanical properties of hyperelastic membranes. The inflation tension test for inflating circular specimens [1,2], the equibiaxial planar tension test for square specimens [3], and the radial tension test for circular specimens [4] are the three most frequently used equibiaxial tension test techniques. The schematic diagrams of the tension tests are shown in Figure 1.

The inflation tension is first employed for the equibiaxial tension test after being motivated by the phenomenon of an inflated balloon expanding into a balloon with equibiaxial deformation. Treloar [5] developed a rubber membrane model to explore the shape and strain distribution on the balloon surface with varying degrees of inflation. Rivlin et al. [6] adopted data collected on the top of the inflated diaphragm as homogeneous two-dimensional tensile data. Adkins et al. [7] are the first to conduct extensive theoretical research on strain energy density functions (SEDFs) in the Neo-Hookean and Mooney forms. Hart-Smith et al. [8] investigated the inflation tension test further and confirmed previous findings using an exponential hyperbolic model. Because of its simple form and ease of use, the inflation tension apparatus is the favored choice for equibiaxial tension tests on materials such as EAP membrane materials [9,10].

Following the pioneering work of Lanir and Fung on the rabbit skin test in 1974 [11], the equibiaxial planar tension test has become widely utilized in mechanical property studies of soft tissue materials [12]. This is particularly applicable to specimens that require processing, where strains are small. Equibiaxial planar tension testing is also commonly employed for polymer materials such as rubber, which exhibit hyperelasticity characteristics [13,14]. The accuracy of experimental results under equibiaxial planar tension may be compromised by non-uniform deformation near the corner of the tension test [15]. Blatz et al. [16] added clips at the four corner points of a square membrane specimen for clamping and stretching; Obata et al. [17] modified the corner chucks to lessen the non-uniformity of specimen deformation at the corners; Jacobs et al. [18] used finite element software to further reveal the stress concentration and stress shielding phenomena in biaxial planar tension. The equibiaxial planar tension apparatus is preferred for its controllability, high reliability, and widespread availability despite its large size and intricate structure.

Unlike inflating a thin elastomer membrane using air pressure, the disc specimen can achieve an equibiaxial state of stress and strain in a plane by applying a constant load around its perimeter. Usually, a specific radial tension apparatus has been employed for circular sheet specimens with cuts and punched holes [4,19], which effectively achieves the pure strain state required for hyperelastic constitutive models.

Despite the widespread use of various equibiaxial tension test methods, no comprehensive comparative analysis of these methods and their accuracy has been published due to their complexity. Since it requires fewer physical prototypes and experiments during the design cycle, FEM is now widely utilized to develop and optimize complex processes or products. There is no question that using the FEM to assess the equibiaxial tension test method is an achievable choice.

VHB acrylic series hyperelastic EAP elastomers provided by 3 M™ exhibit remarkable characteristics of large elasticity and strain energy density, making them highly promising for applications in high-tech fields such as soft robotics [20,21], flexible sensors [22,23], and other dielectric elastomer transducers [24,25]. The property of equibiaxial tension is essential for conducting relevant application research. This study uses the FE software Abaqus 6.14 to model the three basic equibiaxial tension test methods and investigate the mechanical behavior of hyperelastic EAP membranes under quasi-static conditions.

The impact of apparatus structure and specimen geometry parameters on experimental accuracy and other performance factors is also addressed. Additionally, the concept of tensile efficiency is introduced to analyze non-uniform deformation in equibiaxial planar tension and radial tension tests. The stress equation is utilized to explain the reason for the limited deformation simulation range of inflation tensions. This paper’s conclusion can guide the selection and application of equibiaxial tensile testing.

## 2. Constitutive Model of Hyperelastic Membrane Based on Equibiaxial Tension

Various hyperelastic models are available for rubber-like materials [26]. A hybrid model that combines the Flory–Erman and Arruda–Boyce models accurately predicts Treloar’s classical data across all stretching ranges and deformation states [27]. Physically based constitutive models can better account for hyperelasticity, visco-hyperelasticity, and damage phenomena [28]. Recently, these models have also been employed to represent the mechanical behavior of hydrogels and their composites [29,30], while refined hyperelastic models such as the hyperfoam model can accurately describe polymeric foam fabricated through 3D printing [31]. It is worth noting that factors like temperature and aging time may influence the model parameters [32]. The following section will provide a brief introduction to three typical models.

### 2.1. Mooney–Rivlin Model

For incompressible materials, the SEDFs can also be considered as a function of two strain invariants [26,33]:

(1) $$W=\sum_{k+l=1}^{N}C_{kl}(I_{1}-3{)}^{k}(I_{2}-3{)}^{l}$$

where *C_kl_* is the Mooney–Rivlin material parameter and *N* is the model order. In practical application, the first two terms of its power series are usually taken, i.e.,

(2) $$W=C_{10}(I_{1}-3)+C_{01}(I_{2}-3)$$

here *I*_1_ and *I*_2_ are the strain invariants of the Cauchy–Green deformation tensor, which are determined by the stretch ratios *λ_i_* (*i* = 1, 2, and 3) in three principal directions. The stretch ratio is the ratio of the geometric dimension of the stretched specimen to the original one in those directions.

(3) $$I_{1}=\lambda_{1}^{2}+\lambda_{2}^{2}+\lambda_{3}^{2}$$

(4) $$I_{2}=\lambda_{1}^{2}\lambda_{2}^{2}+\lambda_{2}^{2}\lambda_{3}^{2}+\lambda_{3}^{2}\lambda_{1}^{2}$$

### 2.2. Yeoh Model

In the SEDFs formula of Mooney–Rivlin, if only invariant *I*_1_ is partially expanded, the typical third-order Yeoh SEDFs can be obtained [34]:

(5) $$W=C_{10}(I_{1}-3)+C_{20}(I_{1}-3{)}^{2}+C_{30}(I_{1}-3{)}^{3}$$

### 2.3. Ogden Model

Ogden removed the restriction that the SEDFs are an even power of the stretch ratio and proposed a SEDFs in the series form [35]:

(6) $$W\;=\sum_{k=1}^{N}\frac{\mu_{k}}{\alpha_{k}}\left(\lambda_{1}^{\alpha_{k}}+\lambda_{2}^{\alpha_{k}}+\lambda_{3}^{\alpha_{k}}-3\right)$$

where *μ_k_*, *α_k_* are the material parameters. The above-mentioned Ogden SEDFs usually take another form [1,9,10]:

(7) $$W\;=\sum_{k=1}^{N}\frac{{2\mu }_{k}}{\alpha_{k}^{2}}\left(\lambda_{1}^{\alpha_{k}}+\lambda_{2}^{\alpha_{k}}+\lambda_{3}^{\alpha_{k}}-3\right)$$

This formula is also used in the FEM software, Abaqus 6.14. It is the same as the original formula, with only a formal difference. For incompressible materials, with the relation *λ*_1_*λ*_2_*λ*_3_ = 1, the SEDFs can be simplified.

### 2.4. Mechanical Behavior Based on Equibiaxial Tension

According to the SEDFs, the principal Cauchy stress *σ_i_* (*I* = 1, 2, and 3) can be derived as follows:

(8) $$\sigma_{i}=\lambda_{i}\frac{\partial W}{\partial \lambda_{i}}-P_{h}$$

where *P*_h_ is the hydrostatic pressure determined by the dynamic boundary condition. Since the third dimension (thickness) is much smaller than the other two planar dimensions, it can be well approximated to a planar tensile state with *σ*_3_ = 0, and the expressions of the stress in two principal directions under the condition of equibiaxial tension can be deduced:

(9) $$\sigma_{1}=\sigma_{2}=\sigma =\lambda_{1}\frac{\partial W}{\partial \lambda_{1}}-\lambda_{3}\frac{\partial W}{\partial \lambda_{3}}$$

The equibiaxial tensile stress *σ* can be derived with the assumption of isotropy and incompressibility, where $\lambda_{1}=\lambda_{2}=\lambda$ and $\lambda_{3}=1/\lambda^{2}$. When the SEDFs of Yeoh or Mooney–Rivlin are used, Equation (9) can also be rewritten directly in terms of *I*_1_ and *I*_2_ as

(10) $$\sigma =2\left(\lambda^{2}-\lambda^{-4}\right)(\frac{\partial W}{\partial I_{1}}+\lambda^{2}\frac{\partial W}{\partial I_{2}})$$

The stress formula for various models of equibiaxial tension can be found by substituting the above-mentioned SEDFs into Equations (9) or (10). The mechanical behavior of hyperelastic materials is typically expressed as the relationship between engineering stress *S* and stretch ratio *λ*, where *S* equals Cauchy stress (also known as real stress) divided by *λ*:

*S* = *σ*/*λ* (11)

The EAP investigated in this study is VHB4910, a commercial double-sided adhesive tape belonging to the acrylic polymer family and commonly used in the production of flexible actuators due to its high deformation capacity under an electric field. The material model parameters are obtained from the literature [9] and presented in Table 1 below.

## 3. Equibiaxial Tension Tests and Their Simulations for Hyperelastic Membrane

In practice, the methods used to derive mechanical behavior from equibiaxial tension tests vary. Therefore, it is necessary to evaluate equibiaxial tensions through simulations. The model updating method, standardized tests, and the multi-response surface method (MRS) can be combined with desirability functions to automatically determine the most appropriate constants for modeling material behavior using FEM [36]. Consequently, we apply the FEM software ABAQUS to simulate the equibiaxial tension methods and select the Ogden model with the parameters listed in Table 1.

### 3.1. Inflation Tension

**Figure 2:**
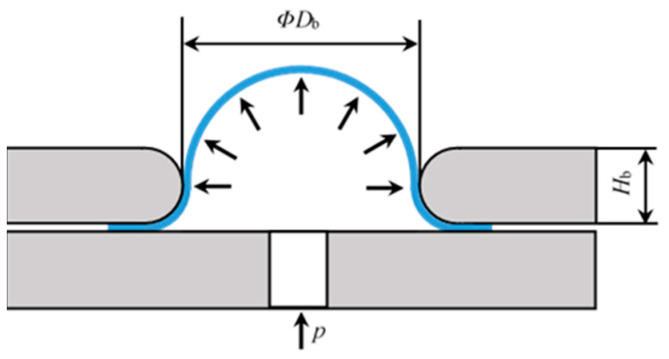
Schematic diagram of inflation tension.

In an inflation tension, the elastic disc diaphragm’s perimeter is held in place during the test while hydraulic oil (or compressed air) is applied to its bottom surface, causing it to expand into a balloon form Figure 1a. The balloon’s symmetry allows for natural equibiaxial stress and strain in its top area. Test equipment commonly uses upper and lower flanges to fix the periphery of the disc diaphragm. Inflation causes the elastic membrane to resemble a balloon through the upper flange hole (Figure 2). The stress and stretch ratio can be calculated using the deformation of an inflated balloon and hydraulic oil or compressed air pressure.

#### 3.1.1. Deformation of Inflation Tension

**Figure 3:**
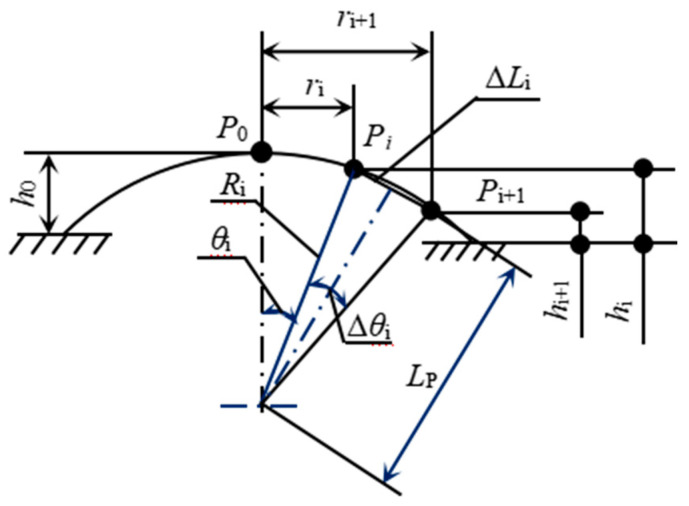
Calculating the geometry of a balloon cap.

In the inflation tension, membrane deformation is typically estimated from sampled points in the deformation zone closer to the vertex of the inflated balloon. During inflation, changes in position of the balloon vertex and surrounding points are recorded and selected for relevance calculation, allowing stretch ratio to be calculated accordingly (Figure 3).

Assuming a balloon-like shape, two observation points situated along the longitude of the inflated surface, $P_{i}$ and $P_{i+1}$, can be utilized for calculation purposes. In the figure, *P*_0_ refers to the vertex, which coincides with the center of the disc diaphragm in its initial state without deformation; *θ_i_* and ${∆\theta }_{i}$ are the central angles for the arc $\hat{{P_{0}P}_{i}}$ and $\hat{{P_{i}P}_{i+1}}$, respectively, Δ*θ_i_* = *θ*_*i*+1_ − *θ_i_*; $h_{i}$ and $h_{i+1}$ are the inflated heights of the points; $r_{i}$, $r_{i+1}$ are the distances from the symmetry axis. Therefore, the longitudinal stretch ratio at a given point $P_{i}$ can be determined for hyperelastic membranes:

(12) $$\lambda_{}^{i}=\frac{R_{i}\Delta \theta_{i}}{\Delta r_{i}^{0}}$$

where *R*_i_ represents the curvature radius at the point *P_i_* and $∆r_{i}^{0}$ denotes the distance between $P_{i}$ and $P_{i+1}$ along the radial direction in their unformed state. According to the geometric relationship illustrated in Figure 3, there are also

(13) $${\left(\Delta L_{i}\right)}^{2}={\left(r_{i+1}-r_{i}\right)}^{2}+{\left(h_{i+1}-h_{i}\right)}^{2}$$

(14) $$\tan(\theta_{i}+\frac{\Delta \theta_{i}}{2})=\frac{h_{i}-h_{i+1}}{r_{i+1}-r_{i}}$$

(15) $$L_{P}=\frac{(r_{i+1}+r_{i})/2}{\sin\left(\theta_{i}+\Delta \theta_{i}/2\right)}$$

where Δ*L_i_* is the chord length in the equation, while $L_{P}$ is the distance between the balloon’s center and the chord. The central angle ${∆\theta }_{i}$ and the curvature radius $R_{i}$ can be calculated in this way:

(16) $$\Delta \theta_{i}=2\arctan(\frac{\Delta L_{i}}{2L_{P}})$$

(17) $$R_{i}=\frac{\Delta L_{i}/2}{\arcsin(\Delta \theta_{i}/2)}$$

When the distance between two observation points is sufficiently short, the equations can be used to approximate and determine the curvature radius *R*_i_ and stretch ratios of the inflated balloon’s surface points. The balloon vertex usually serves as the starting point and then the curvature radius *R*_i_ becomes an average one.

#### 3.1.2. Stress of Inflation Tension

The equibiaxial tensile stress can be determined using the curvature radius *R*, longitudinal stretch ratio *λ*, compressed air (or hydraulic oil) pressure *p*, and membrane thickness *t*, assuming that the inflated form in the inflation tension is “balloon-like” [7]:

(18) $$\sigma =\frac{pR}{2t}=\frac{pR\lambda^{2}}{2t_{0}}$$

where *t*_0_ is the membrane’s initial thickness, and the manufacturer-specified value for the VHB4910 membrane is 1 mm.

#### 3.1.3. Simulation of Inflation Tension

The homogeneity of deformation has a significant impact on the accuracy of inflation tension tests. As a circular diaphragm specimen, the primary factors influencing test results are the structural dimensions of the apparatus, specifically the upper flange’s inflation hole diameter (*D*_b_) and thickness (*H*_b_), which also determine the transition arc radius (*H*_b_/2). Therefore, this simulation focuses primarily on deformation uniformity and how the structural dimensions of the apparatus affect experimental accuracy, with *H*_b_ = 6 mm and *D*_b_ = 54 mm utilized in the simulation.

**Figure 4:**
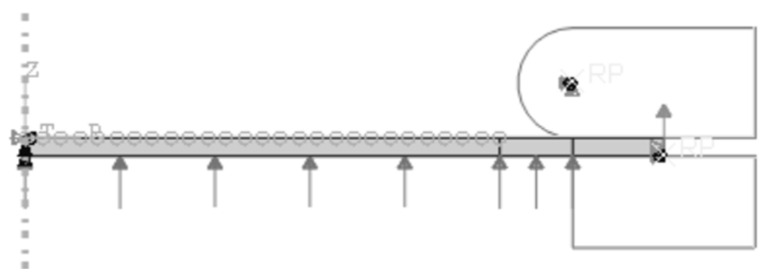
Scheme of the model for inflation tension.

**Figure 5:**
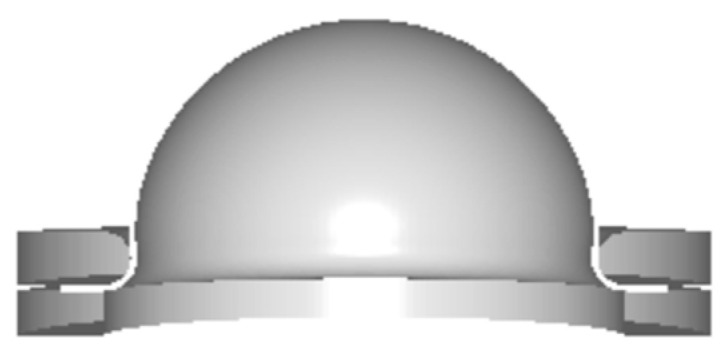
FEM simulation for inflation tension.

The simulation employs a four-node, bilinear, axisymmetric quadrilateral hybrid element (CAX4H) model for the EAP membrane specimen. To satisfy the axial symmetry boundary conditions, a cylindrical coordinate system is adopted, as depicted in Figure 4. Pressure is applied beneath the circular diaphragm, while the upper and lower flanges act as rigid surfaces with frictionless contact during inflation. The membrane’s periphery is fixed, and its displacement is constrained to zero at the midpoint on the right side. The upper flange remains fixed, allowing upward movement of the lower flange along with membrane deformation, and their boundary conditions are applied at the reference points (RPs). In the simulation, the position changes in multiple observation points on the surface of the circular diaphragm, including the center (vertex), are recorded during inflation in order to calculate the curvature radii of the balloon. The stretch ratio and stress can then be derived using the equations mentioned above. The inflation tension simulation is shown in Figure 5.

### 3.2. Equibiaxial Planar Tension Test

#### 3.2.1. Equibiaxial Planar Tension Method

**Figure 6:**
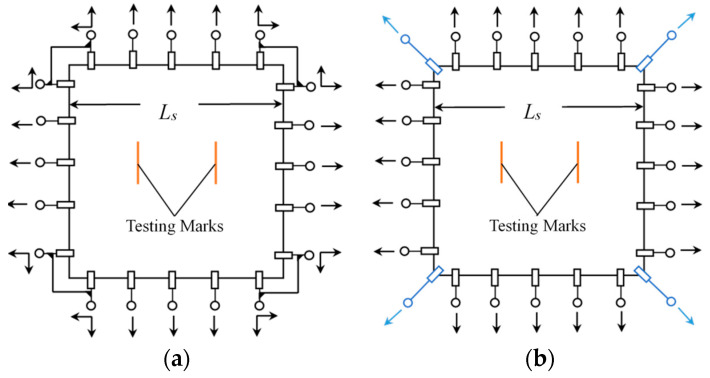
Schematic diagram of an equibiaxial planar tension test: (**a**) two-corner-point-fixed tension; (**b**) single-corner-point tension.

Equibiaxial planar tension is a technique that induces equibiaxial plane deformation by delivering uniform tensile stress (or displacement) to the periphery of a square membrane specimen (Figure 1b). Mechanical clamping is necessary for a realistic experimental setup (Figure 6). To avoid stress concentration and stress shielding [18], it is recommended to use the uniformly distributed multi-point tensile method while keeping the clamping area as small as possible. The concentrated loads applied by the clips along the specimen edge will result in equibiaxial free tension when the tangential displacements of the tensile points along the edge are not constrained. A significant strain arises between the two tensile points near the corner as they are unable to provide sufficient tension for the lateral “fast” movement of other points. This strain can easily lead to specimen rupture when the stretch ratio is sufficiently large. Therefore, two clips on the mutually perpendicular edges close to the corner can be fixed to each other during the stretching process [15], also known as two-corner-point-fixed tension, as seen in Figure 6a.

To improve the uniformity of deformation in the membrane specimen’s corner area, a single-corner-point tension approach can be applied. In the approach, the connecting plate between the two points adjacent to the corner in Figure 6a is eliminated, and four additional clips are introduced at the corners (Figure 6b). Moreover, concentrated forces (or displacements) oriented at a 45° angle to the tensile direction are applied at these corner points (clips) during equibiaxial planar tension testing.

The nominal stress *S* can be derived by measuring and calculating the total axial tensile force *F*, then dividing it by the initial cross-sectional area *A*_0_ of the square membrane.

(19) $$S=\frac{F}{A_{0}}=\frac{F}{L_{s}t_{0}}$$

where *L*_s_ is the square membrane specimen’s edge length. The axial tensile force *F* in the simulation is calculated by summing the tensile forces at each tensile point along the edge and the values of the force component near or at the corner. The displacement of the testing marks can be used to calculate the stretch ratio in equibiaxial planar tension.

#### 3.2.2. Simulation of Equibiaxial Planar Tension

In addition to the clamping technique at (or near) the corner of the specimen, the number of clamping points along its edge also affects the deformation uniformity in the equibiaxial planar tension. The simulation of equibiaxial planar tension is carried out in three cases, each focusing on a square membrane with the same geometric dimensions of 100 mm × 100 mm × 1 mm: (1) two-corner-point-fixed tension with five clips along each edge; (2) single-corner-point tension with five clips along each edge and four clips at the corners; and (3) multi-corner-point tension with four additional clips added to each edge of the specimen based on the two-corner-point-fixed tension. The tensile efficiency *η*^λ^ is defined as the ratio of *λ*^C^ to *λ*^o^ when *λ*^C^ is defined as the central stretch ratio, which is generally estimated at the observation point near the specimen center, and *λ*^o^ is defined as the exterior stretch ratio measured at the tensile point. All *λ*^o^ = 4.6 are used for comparison purposes.

**Figure 7:**
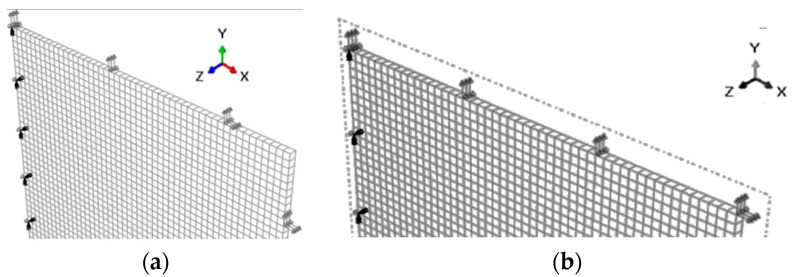
Scheme of the model for equibiaxial planar tension: (**a**) two-corner-point-fixed tension; (**b**) single-corner-point tension.

The hyperelastic membrane FE model is meshed using three-dimensional eight-node hybrid solid elements (C3D8H), and the mesh seeding is controlled with an approximate global size and verified, resulting in a total of 2500 elements. A quarter of the specimen with symmetrical conditions in the left and lower planes is proposed to simulate the equibiaxial planar tension test for a typical square EAP membrane to reduce the computational cost of the provided FE model. The tension mode is also simplified to help in the convergence of the model for simulating practical equibiaxial tension tests. The ramp displacements are eventually applied to the distributed lines rather than the grippers (Figure 7).

In the two-corner-point-fixed tension, the distance between two clamping points held on two perpendicular edges and close to the corner must remain constant during stretching (Figure 7a). The displacement direction of the points is adjusted to 45° from their principal direction when loads are applied to both edges. In the single-corner-point tension, only the displacement direction of the tensile point at the corner is adjusted to 45° (Figure 7b). The tensile points in the multi-point tension are increased based on the two-corner-point-fixed tension. Except for those points located near or at the corners and along the symmetrical axis, the other tensile points in the equibiaxial planar tension are tangential-free.

### 3.3. Radial Tension Test

#### 3.3.1. Radial Tension Method

**Figure 8:**
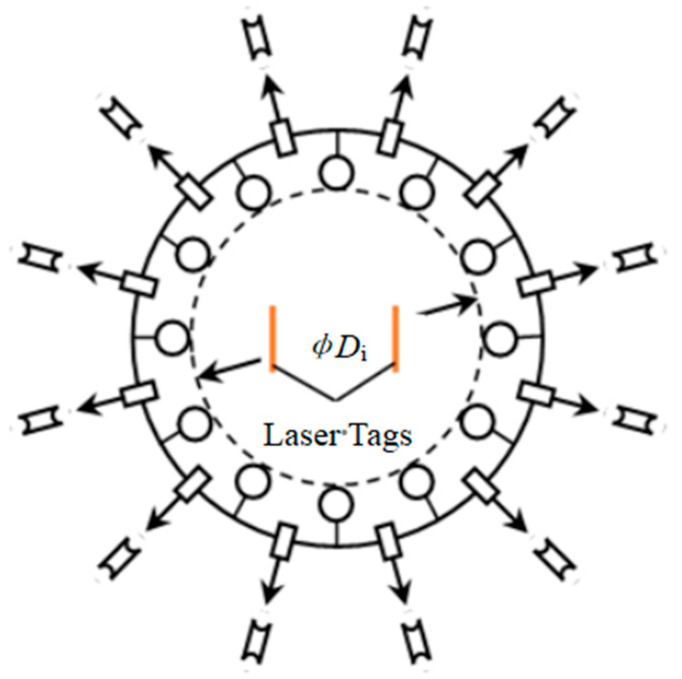
Schematic diagram of radial tension.

To produce equibiaxial strain and determine the mechanical characteristics of a circular diaphragm specimen, the radial tensile test requires applying a uniform radial tensile force (or displacement) around the specimen (Figure 1c). In practical radial tension tests, circular membrane specimens with cuts and punched holes are typically used (Figure 8) to prevent excessive tangential deformation between two grips and avoid ripping of the specimens. The experimental apparatus can achieve the equibiaxial deformation of the specimen by pulling the cable to move the grips evenly distributed radially. It is assumed that the uniform deformation occurs in the range of the circle (*ΦD*_i_) which is externally tangent to the punched holes, and the testing marks in Figure 8 are usually used for strain measurement during tension tests. The nominal stress *S* of equibiaxial radial tension is

(20) $$S=\frac{F}{\pi *D_{i}*t_{0}}$$

where *F* is the sum of the radial tensile forces at each grip. In practice, a laser non-contacting extensometer can be used to measure the strain on the surface of the specimen away from the grips through two laser tags placed on the surface [19].

#### 3.3.2. Radial Tension Simulation

**Figure 9:**
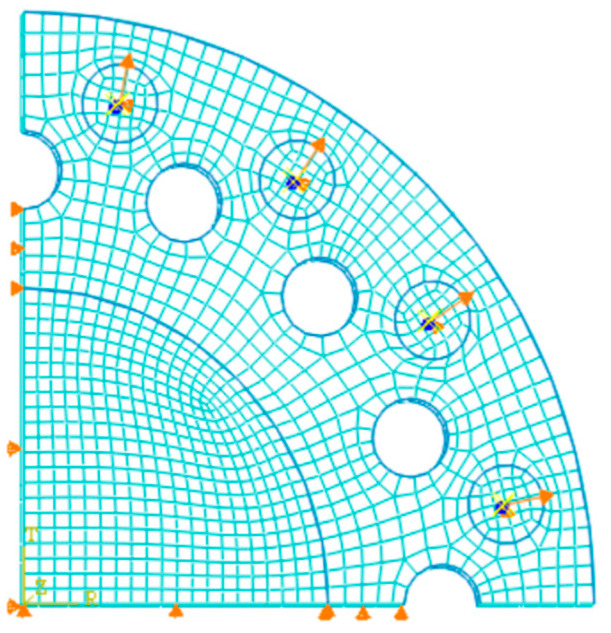
Schematic diagram of the model for radial tension.

The geometrical structure of the circular specimen plays an important role in ensuring test accuracy in radial tension. As a result, the cut number and hole diameter are the main parts of the simulation analysis. Initially, specimens with the same contour dimension (*Φ* 75 mm × 1 mm, *D*_i_ = 65 mm) and punched hole diameter (*Φ* 5 mm) but with 12, 16, and 24 cuts are chosen for modeling. Three-dimensional eight-node hybrid solid elements (C3D8H) are utilized to mesh the FE models. Similarly, to reduce the number of elements and simplify the computation, a 1/4-symmetric membrane specimen is taken for analysis, with the boundary conditions set to axis symmetry (see Figure 9). The round areas on the stretch bands are used to simulate the grips [19], and a uniformly distributed radial displacement load is applied to the centers of the surface. In the simulation, the stretch ratio calculated from the center of the gripping round area is set as *λ*^o^ = 4.5.

## 4. Results and Discussion

Stress concentration and stress shielding, typically arising from non-uniform deformation, are the primary factors contributing to a decrease in test accuracy. Hence, it is imperative to engage in simulation-based discussions regarding deformation in various tests and employ simulations to demonstrate other experimental properties. Additionally, the accuracy of the tests can be validated by comparing stresses obtained from different testing simulations with theoretical calculation values derived using the aforementioned formula for square membranes with an Ogden material model and parameters sourced from the literature [9].

### 4.1. Equibiaxial Inflation Tension

#### 4.1.1. Deformation Profile

**Figure 10:**
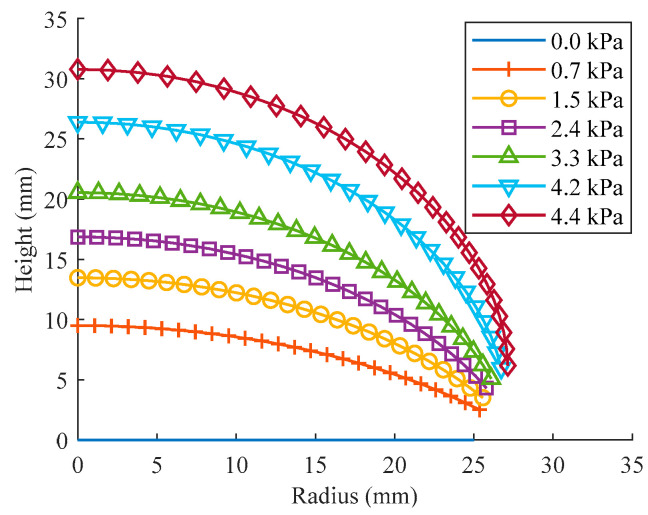
Surface profiles of the balloon from the simulation of inflation tension with Ogden model.

Starting from the circular membrane’s symmetry center in its unformed condition, a series of observation points of equal distance (1 mm) are taken along the radial direction to record their positions during the inflation, and then the profile curves of the balloon can be obtained. Figure 10 illustrates how the curvatures of the balloon surface change during inflating. The curvatures of the balloon’s surface at the observation point are uneven; they are flatter in the top region and sharper outward.

**Figure 11:**
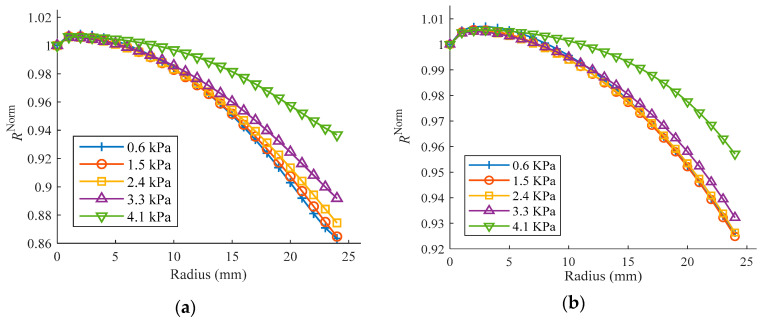
Normalized curvature radii (*R*^Norm^) from the simulation of inflation tension with Ogden model: (**a**) at uniformly distributed observation points; (**b**) when starting point is fixed at the vertex.

By utilizing Equation (17) and the simulation position information for the observation points on the surface of the inflated balloon, the curvature radii can be calculated. To conveniently understand the profile deformation of the balloon at various pressures, the curvature radii are normalized by the radius of the point closest to the vertex (1 mm away from the symmetry axis), as shown in Figure 11. The curvature radii in Figure 11a are determined based on adjacent observation points. However, if the starting point is fixed at the vertex, the calculated curvature radii represent an average value (Figure 11b).

As can be seen from the figure, the curvature radius of the profile reduces progressively along the longitude, except for a tiny fluctuation at the beginning point caused by the simplified simulation model, and the discrepancy of the radii also dwindled as the inflation pressure increased. In Figure 11a, as the pressure increases from 0.6 kPa to 4.1 kPa, there is an obvious decrease in the difference of curvature radii at the outer observation point (located 24 mm away from the symmetry axis) with respect to the curvature radius at the vertex, decreasing from approximately 13.7% to 6.3%. If the studied segment’s starting point is fixed at the vertex (Figure 11b), a similar variation tendency is noticed in the average curvature radii, but the overall difference is reduced due to the averaging effect.

**Figure 12:**
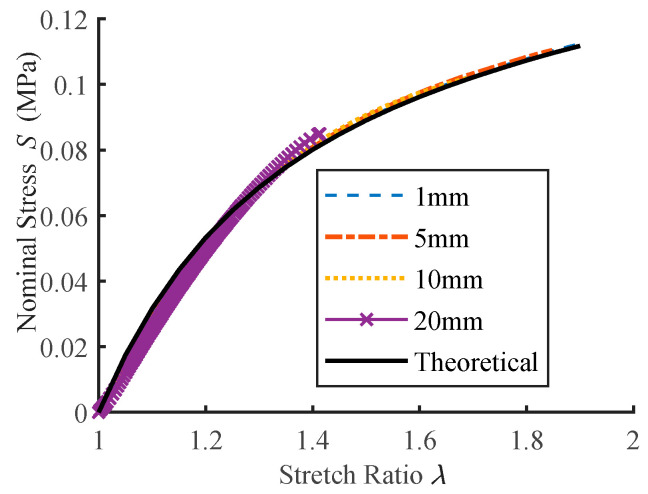
*S*-*λ* curves at different observation points from the simulation of inflation tension with the Ogden model.

The inflated ball’s non-uniform deformation and profile error will result in an incorrect characteristic curve in terms of nominal stress against stretch ratio. In the unformed state, four observation points at various distances (1, 5, 10, and 20 mm away from the disc membrane’s center, respectively) are selected for analysis. Calculated *S*-*λ* curves are then compared to the results of theoretical calculations (Figure 12). From the figure, it is evident that the deviation increases as the observation point moves away from the center; moreover, due to less deformation, the effective experimental range significantly reduces at farther points. In Figure 12, when inflation pressure reaches 4.1 kPa and the observation point is 20 mm away from the center, the calculated stress on the curve exceeds the theoretical result by 4.7%, but the actual deformation is less than half of that at the vertex due to non-uniformity.

#### 4.1.2. Influence of Structure Dimensions

The main structure dimensions of the inflation tension apparatus include the thickness *H*_b_ of the upper flange and the diameter *D*_b_ of the inflation hole (Figure 2). Accordingly, three simulation models are proposed for the analysis:

- Original model: The test apparatus model has the same dimensions as the original structure.
- Thick flange model: According to the original model, the thickness of the upper flange is doubled.
- Small hole model: Based on the original model, the inflation hole of the structure is halved.

**Figure 13:**
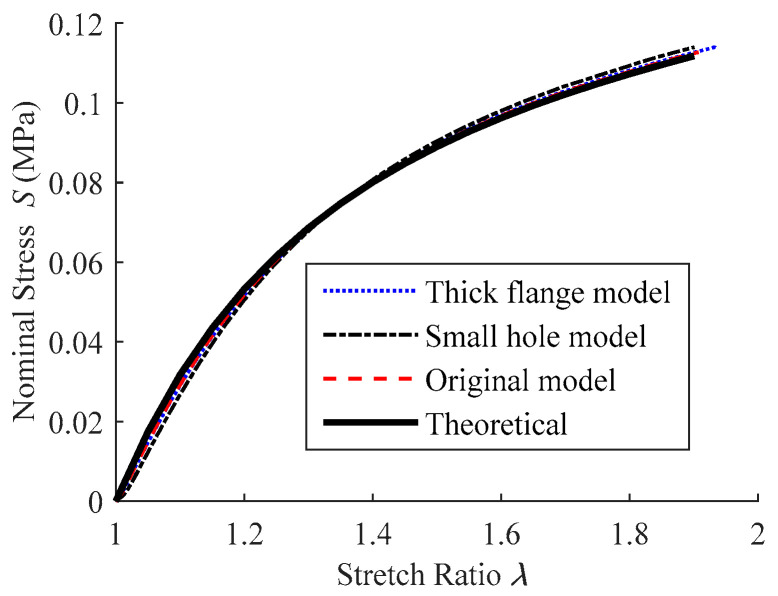
Comparison of *S*-*λ* curves from the simulation with different structure dimensions in the experimental apparatus.

The *S*-*λ* curves derived from the aforementioned models are depicted in Figure 13, along with the theoretically calculated result for comparison. The four curves are near each other throughout the range of stretch ratio, with the biggest discrepancy occurring in the experimental apparatus with a small hole due to nonuniform deformation. When *λ* ≈ 1.9, the stresses in the small hole model are somewhat higher than the theoretical calculation, but not more than 2%. Within the stretch ratio range, it can be concluded that the impact of apparatus parameters on test accuracy may be ignored as long as the calculated data are sampled from the uniformly deformed area near the vertex of the inflated balloon.

**Figure 14:**
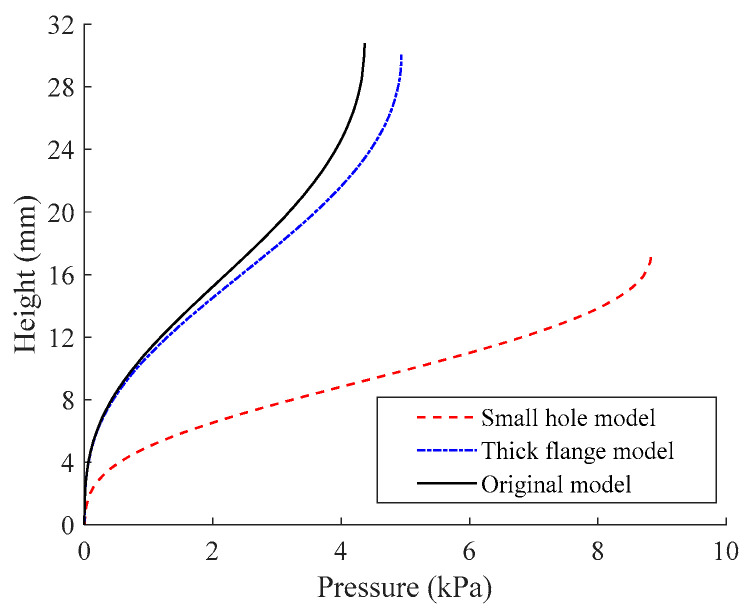
Deformation height from the simulation of inflation tension with different structure dimensions.

According to the aforementioned models with different structural dimensions, the inflation heights during the inflation tension test can also be determined (Figure 14). The figure illustrates that as the stretch ratio reaches *λ* ≈ 1.9 at the vertex, a small inflation hole in the apparatus will cause a reduction in the volume of the inflated balloon. By reducing the diameter of the inflation hole by 50%, the deformation height will be reduced by half to 17.2 mm, while the required air pressure will be doubled to about 8.83 kPa.

Equation (18) implies that the inflation pressure is inversely proportional to the inflated balloon’s radius of curvature. The curvature radius at the vertex of the small hole model is about 13.6 mm when *λ* = 1.9, which is roughly half the radius of the original model. As a result, the necessary air pressure must be raised. Additionally, Figure 14 illustrates how thickening the upper flange will lead to less deformation and more air pressure. The inflation hole will be slightly smaller as a result of thickening the flange, which has the same effect as reducing the inflation hole.

#### 4.1.3. Deformation Range during Inflation Tension Simulation

The research investigation ends when the equibiaxial stretch ratio reaches approximately 1.9 due to convergence issues and error reporting in ABAQUS, resulting in a limited range of inflation that deviates from practical results. These issues are primarily caused by the material model and its parameters. Substituting the equibiaxial tensile stress from Equation (10) into Equation (18) gives

(21) $$p=\frac{2\sigma t}{R}=\frac{4t_{0}(1-\lambda^{-6})}{R}(\frac{\partial W}{\partial I_{1}}+\lambda^{2}\frac{\partial W}{\partial I_{2}})$$

The SEDF is expressed differently on the right side of Equation (21). Because the components *R*, $\partial W/\partial I_{1}$, and $\partial W/\partial I_{2}$ in the equation are functions of the equibiaxial stretch ratio *λ*, the equation has numerous theoretical solutions. Adkins et al. [7] observed that when the parameter $\Gamma =C_{01}/C_{10}$ > 0.21, the air pressure increases monotonically with inflation; when *Γ* < 0.21, the local extreme points will appear in the pressure-deformation equation, and the extreme *p* is obtained when *λ* = 1.84. In the inflation tension simulation, a maximum equibiaxial stretch ratio *λ* of approximately 1.90 can be achieved, which is close to the theoretical value.

In this study, the Moony–Rivlin model is used to re-fit the nominal stress and stretch data that are obtained from Ogden material parameters [9] (see Table 1). According to the Moony–Rivlin model parameters, it is found that *Γ* = 0.0038, which conforms to the latter condition (*Γ* < 0.21), results in multiple deformation solutions at local extreme pressure and diverges in the simulation analysis. Therefore, when applying FE software to analyze the inflation deformation of hyperelastic EAP membranes, we should pay attention to the material model and its parameters, which will affect the analysis range of deformation.

### 4.2. Equibiaxial Planar Tension Simulation

#### 4.2.1. Strain Distribution

**Figure 15:**
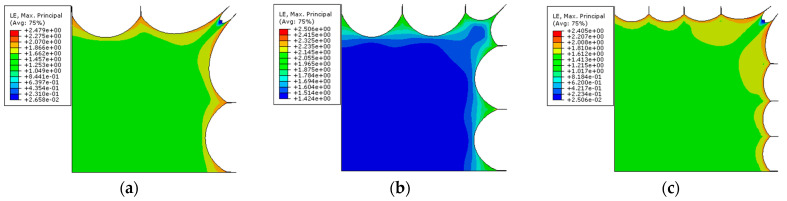
Contour maps of principal strain: (**a**) two-corner-point-fixed tension; (**b**) single-corner-point tension; and (**c**) multi-point tension with two corner points fixed.

Figure 15 depicts three strain contour maps for various tensions. The larger strain near the tensile point can be seen in the equibiaxial planar tension, while stress shielding occurs in the two-corner-point-fixed tension (Figure 15a,c). The single-corner-point tension (Figure 15b), on the other hand, efficiently removes stress shielding in the corner zone and improves overall stress distribution.

#### 4.2.2. Deformation along Symmetry Axis

**Figure 16:**
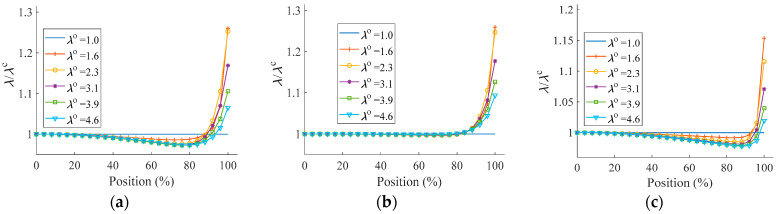
Deformation along the symmetry axis in equibiaxial planar tension: (**a**) two-corner-point-fixed tension; (**b**) single-corner-point tension; and (**c**) multi-point tension with two corner points fixed.

The non-uniformity of deformation in the equibiaxial planar tension affects the precision of the derived stretch ratio, resulting in lowered experimental accuracy. Along the symmetry axis, a series of observation points are allocated, and their positions are stated as the ratio of their geometry position from the center to the length of the investigated specimen ($L_{s}/2$) in an unformed state. The stretch ratio *λ*^c^ of the section closest to the center can be employed to represent the specimen’s stretch ratio when establishing the *S*-*λ* relationship. The stretch ratios obtained from the observation points are then normalized using *λ*^c^, i.e., *λ*/*λ*^c^. As a result, Figure 16 can be used to illustrate the relative axial deformation in various equibiaxial planar tensions.

The figures show that the stretch ratios fluctuate in the axial direction and decrease slightly at first, with an amplitude of no more than 5%; stretch ratios rapidly grow due to pulling at the tensile points after exceeding about 80% of the entire axial length and reach a maximum at the tensile point. The overall deformation of the single-corner-point tension (Figure 16b) is more uniform, and the amplitude of the strain fluctuation is less than 1% within 80% of the entire axial length; the strain fluctuation at the tensile point will decrease as the number of tensile points increases in the two-corner-point-fixed tension (Figure 16c). The adequacy of stretch calculation can also be inferred when sampled deformation data are obtained from less than 20% of the entire length in the two-corner-point-fixed tension (Figure 16a,c) and about 80% in the single-corner-point tension (Figure 16b). The same results can be drawn when the tensile point does not pass through the symmetry axis, but the deformation variation trend is reversed.

#### 4.2.3. The Relationship between Stress and Stretch Ratio in Equibiaxial Planar Tension

**Figure 17:**
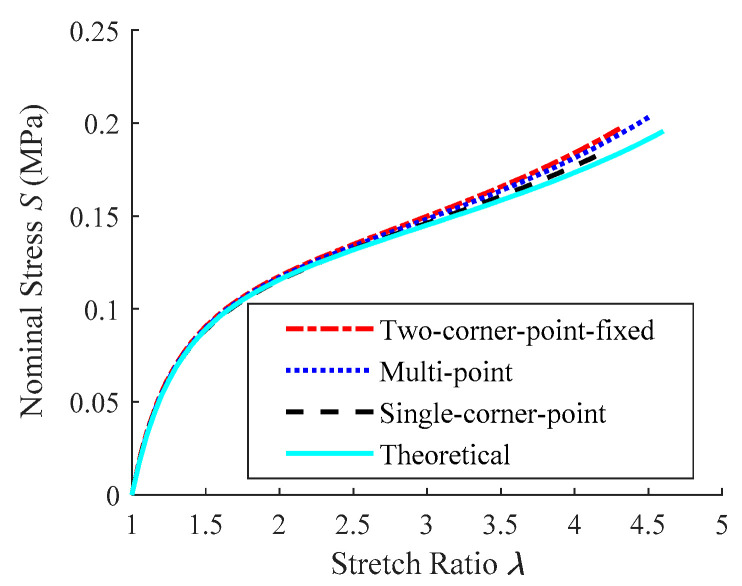
Comparison between the *S*-*λ* curves obtained from the simulation and theoretical calculation.

Add the tensile forces at each tensile point, including the force component at or near the corner point, and then utilize Equation (19) to calculate the nominal stress. The *S*-*λ* curves of the hyperelastic membrane can be determined using the above-mentioned stretch ratio *λ*^c^ as the stretch ratio of the specimen under different tension methods (Figure 17).

The *S*-*λ* curves derived from the three equibiaxial planar tension tests are consistent with the theoretical calculation in the figure, and the discrepancy grows slightly when the stretch ratio increases due to distortion. Because the overall deformation is relatively uniform, the simulation result in single-corner-point tension is the closest to the theoretical calculation, and the stress error is about 2.1% when *λ* = 4. Multi-point tension with two corner points fixed yields the best tensile efficiency, *η*^λ^ ≈ 98.1%, when *λ*^o^ = 4.6 due to the increased number of tensile points and sufficient deformation of the specimen. Table 2 also presents stress error (when *λ* = 4) and tensile efficiency *η*^λ^ (when *λ*^o^ = 4.6) for three types of planar tension.

### 4.3. Radial Tension

**Figure 18:**
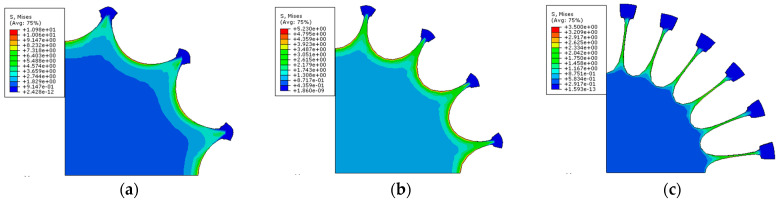
Mises stress contour maps under radial tension: (**a**) 12-cut; (**b**) 16-cut; and (**c**) 24-cut.

The Mises stress contour maps obtained from the radial tension simulations for three different types of specimens are presented in Figure 18. These figures demonstrate that the stress distribution is uniform over a significant area surrounding the center of the circular membrane. When there are more cuts, the boundaries of the stretch band are subjected to substantial stress, whereas fewer cuts result in higher stress levels along the transition arc. Excessive stress can cause rupture or material failure, while excessive deformation reduces tensile effectiveness. Therefore, the strength, accuracy, and tensile efficiency of the number of cuts should be carefully weighed.

#### 4.3.1. Deformation in Radial Tension

To further investigate the homogeneity of the stretch ratios in radial tension, a number of observation points that are uniformly spaced along the symmetry axis from the center of the circular membrane to the circle *D_i_* that is tangent to the punched hole are chosen. Likewise, each point’s position is expressed as a percentage of the total observation length (*D_i_*/2).

**Figure 19:**
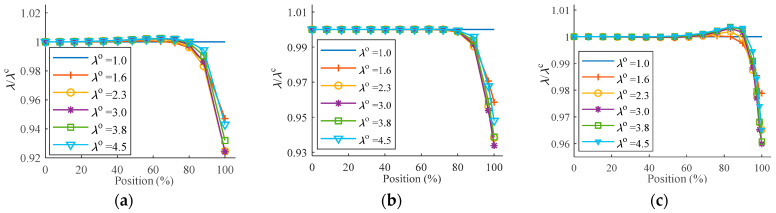
Deformation in radial tension: (**a**) 12-cut; (**b**) 16-cut; and (**c**) 24-cut.

The stretch ratio calculated from each point is normalized by the stretch ratio calculated from the center (*λ/λ*^C^). The stretch ratio is uniform throughout the radial distance of about 80% from the center to the inner tangent circle *D*_i_, as indicated in Figure 19, especially in the specimen with a 16-cut (Figure 19b). The stretch ratio increases slightly from the center outward at first, but the amplitude is very modest, less than 0.5%, and then abruptly decreases. In contrast to the deformation variations induced by equibiaxial planar tension of the rectangular membrane, the sampled points in the simulation are taken along the direction of the symmetry axis between the two grips, resulting in shrinkage in the stretching process and leading to a smaller stretch ratio at the outer end. With a reduction in the number of cuts, the effects of shrinkage become more pronounced. It can be observed from Figure 19 that at *λ*^o^ = 4.5, the shrinkage in the specimen with 12 cuts approaches 8% (Figure 19a), while that of the specimen with 24 cuts is less than 4% (Figure 19c).

#### 4.3.2. The Relationship between Stress and Stretch Ratio in Radial Tension

**Figure 20:**
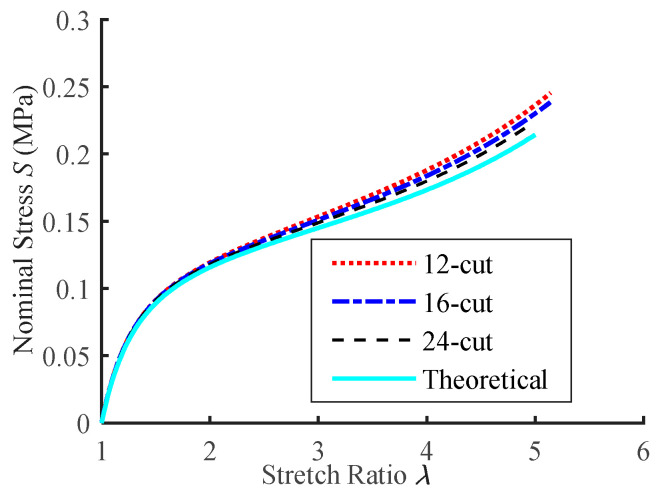
Stress-strain relationships in radial tension.

By summing the tensile forces of the radial points, the nominal stress can be calculated using Equation (20). The stretch ratios are obtained from an observation point near the center, and the *S*-*λ* curves for hyperelastic membrane specimens with varying numbers of cuts are depicted in Figure 20.

It can be seen from the figure that the *S*-*λ* curves of the EAP membrane materials agree well with the theoretical calculation result. The more cuts there are, the more uniform the deformation of the specimen and the more accurate the *S*-*λ* curves. When *λ* = 4, the 24-cut specimen has the lowest stress error, less than 4% of the theoretical result.

The “pull” effect on the center area of the specimen with fewer cuts becomes noteworthy due to high stress in the transition arc between the grips, resulting in increased tensile efficiency, where the stretch ratios of 12-cut and 16-cut specimens even exceed that from the gripping points. Table 3 presents the calculated tensile efficiency *η*^λ^ (*λ*^o^ = 4.5), along with the corresponding stress errors (*λ* = 4).

#### 4.3.3. Influence of the Punched Hole Size

The punched hole at the end of the cut is utilized to reduce stress concentration and prevent rupture. As demonstrated in Table 3, reducing the diameter of the hole will increase the tensile stress along the transition arc, which results in nonuniform deformation and a slight increase in the stress error. As for the tensile efficiency *η*^λ^, although increasing the number of cuts can thin the stretch bands and reduce the tensile efficiency, decreasing the diameter of the punched hole can increase the stresses around the root of the cut, strengthen deformation, and increase the efficiency. The above two factors have a hedging effect on tensile efficiency.

## 5. Conclusions

In this paper, FEM is utilized to model and validate three typical equibiaxial tension methods, and the influences of the apparatus structure and specimen geometry parameters on the test results are also analyzed. The following conclusions can be drawn:

The sample point for calculating deformation in the inflation tension test should be positioned as close to the vertex of the balloon as possible, ensuring accurate results for the characteristic curve and expanding the effective experimental range. The structure dimensions of the test apparatus have minimal impact on the final test accuracy within the simulated deformation range, although the large inflation hole can reduce the required test pressure, whereas the tiny hole possesses a low deformation height. It is worth noting that the range of EAP simulation analysis can be influenced by the material model and its parameters, with a maximum limit of *λ* ≈ 1.9 under certain conditions.

Among the two equibiaxial planar tension methods proposed in this paper, the deformation in the single-corner-point tension exhibits a higher level of uniformity, and the characteristic curve obtained closely approximates the theoretically calculated result with a minimal stress error of 2.1% when *λ* = 4. Increasing the tensile points can make the overall deformation of the specimen more sufficient, thus obtaining a higher tensile efficiency *η^λ^*, which also helps to reduce the force error.

In the radial tension test, a decrease in stress error is observed with an increase in the number of cuts and larger punched hole diameters. By utilizing a 24-cut specimen with a punched hole diameter of 5 mm, it is possible to achieve a minimal stress error of approximately 3.83%. Increasing the diameter of the punched hole will reduce the stress in the transition arc between cut edges. However, employing a larger hole with more cuts results in a smaller stretch band, leading to severe tensile deformation and strength failure of the membrane. Additionally, excessive cuts may pose fabrication challenges. It should be noted that excessive deformation of the stretch band can lead to reduced tensile efficiency; however, this effect can be mitigated by increasing the number of cuts and reducing the diameter of punched holes on specimens.

The conclusions derived in this paper can provide a useful guide for choosing an equibiaxial tension test method, designing an apparatus or specimen geometry, and evaluating the accuracy of the experiment. Although this paper focuses on the three typical equibiaxial tension methods, it can easily be extrapolated to other equibiaxial tension tests.

## Figures and Tables

**Table 1:** Material model parameters.

The Second-Order Ogden Model (Abaqus Form) [9]		The First-Order Mooney–Rivlin Model ^a^	
*μ*_1_ (kPa)	64.7	*C*_01_ (kPa)	21.56
*α*_1_	1.39689		
*μ*_2_ (kPa)	0.0457	*C*_10_ (kPa)	0.0815
*α*_2_	5.8638		

^a^ The parameters of the first-order Mooney–Rivlin model are obtained by refitting the data from the second-order Ogden model.

**Table 2:** Characteristics of equibiaxial planar tension.

Tension Method	Two-Corner-Point-Fixed Tension	Single-Corner-Point Tension	Multi-Point Tension
Stress error (%)	6.2	2.1	4.6
*η*^λ^ (%)	93.9	91.4	98.1

**Table 3:** Comparison of stress error and tensile efficiency.

Specimen	12-Cut	16-Cut	24-Cut
Stress error (%)	8.48(8.59)	6.05(8.46)	3.83(4.65)
*η^λ^* (%)	101.52(98.03)	101.12(99.55)	96.72(100.04)

Note: The data in brackets are from the punched specimens with the diameter reduced by half.

## Data Availability

Data will be available upon request.
